# Does multi-way, long-range chromatin contact data advance 3D genome reconstruction?

**DOI:** 10.1186/s12859-023-05170-x

**Published:** 2023-02-24

**Authors:** Adam B. Olshen, Mark R. Segal

**Affiliations:** 1 Department of Epidemiology and Biostatistics and Helen Diller Family Comprehensive Cancer Center, University of California, San Francisco, CA USA; 2 Department of Epidemiology and Biostatistics, University of California, San Francisco, CA USA

**Keywords:** Multi-dimensional scaling, SPRITE, Conformation capture, Pairwise distance, Procrustes alignment

## Abstract

**Background:**

Methods for inferring the three-dimensional (3D) configuration of chromatin from conformation capture assays that provide strictly pairwise interactions, notably Hi-C, utilize the attendant contact matrix as input. More recent assays, in particular split-pool recognition of interactions by tag extension (SPRITE), capture multi-way interactions instead of solely pairwise contacts. These assays yield contacts that straddle appreciably greater genomic distances than Hi-C, in addition to instances of exceptionally high-order chromatin interaction. Such attributes are anticipated to be consequential with respect to 3D genome reconstruction, a task yet to be undertaken with multi-way contact data. However, performing such 3D reconstruction using distance-based reconstruction techniques requires framing multi-way contacts as (pairwise) distances. Comparing approaches for so doing, and assessing the resultant impact of long-range and multi-way contacts, are the objectives of this study.

**Results:**

We obtained 3D reconstructions via multi-dimensional scaling under a variety of weighting schemes for mapping SPRITE multi-way contacts to pairwise distances. Resultant configurations were compared following Procrustes alignment and relationships were assessed between associated Procrustes root mean square errors and key features such as the extent of multi-way and/or long-range contacts. We found that these features had surprisingly limited influence on 3D reconstruction, a finding we attribute to their influence being diminished by the preponderance of pairwise contacts.

**Conclusion:**

Distance-based 3D genome reconstruction using SPRITE multi-way contact data is not appreciably affected by the weighting scheme used to convert multi-way interactions to pairwise distances.

## Background

The task of reconstructing the three-dimensional (3D) configuration of chromatin within the eukaryotic nucleus from pairwise contact data, notably Hi-C [1–3], is motivated by (at least) three considerations. First, such architecture is critical to an array of cellular processes, particularly transcription, but also, for example, memory formation [4]. Second, armed with such an inferred configuration, it is possible to superpose genomic attributes, enabling biological insights not accessible from a primary Hi-C contact matrix readout. Examples here include the identification of gene expression gradients and co-localization of virulence genes in the malaria parasite [5], the impact of spatial organization on double strand break repair [6], and the elucidation of ‘3D hotspots’ corresponding to (say) overlaid ChIP-Seq transcription factor extremes that can reveal novel regulatory interactions [7]. Third, despite notable gains in imaging methodologies [8], direct access to structure is yet to enjoy the resolution and uptake provided by Hi-C assays.

This set of factors has led to a wealth of 3D reconstruction algorithms: a recent review [9] identified over 30 methods and there have been numerous additions in subsequent years. However, the very notion of ‘a’ 3D reconstruction is simplistic as genomes are dynamic and variable with structural differences by organism, tissue, cell-type, cell-cycle, and cell. Hi-C experiments are frequently performed on large, synchronized, cell-type specific populations so that a resulting reconstruction is interpreted as providing a consensus configuration. The emergence of single cell Hi-C (scHi-C; [10, 11]) has enabled dissection of inter-cellular structural variation, at the expense of yielding appreciably sparser data.

Another component of structural variation is allelic: in diploid organisms maternal and paternal homologs can adopt differing configurations. This poses difficulties for reconstruction algorithms since Hi-C data is generally unphased so that it is ambiguous whether contacts are intra- or inter- homolog. Until recently, these concerns have been ignored, but novel approaches attempt to resolve identifiability issues by either prescribing (strong) assumptions and/or invoking additional data sources (detailed next) [12, 13]. We do not address these aspects here, but note that the concerns can be sidestepped if Hi-C data is phased, HiCHap [14] being an accurate tool for this purpose.

The additional data deployed by Belyaeva et al. [13] to resolve allelic ambiguity and obtain diploid 3D reconstructions derives from multi-way (as opposed to pairwise) contact assays, such as split-pool recognition of interactions by tag extension (SPRITE, [15]) or genomic architecture mapping (GAM, [16]). In this paper we explore the use of such data as the direct *basis* for effecting 3D reconstruction, a task yet to be undertaken as far as we are aware. The rationale for so doing derives from key features of SPRITE data as they contrast with standard Hi-C and relate to nuclear chromatin organization, specifically, high-order multi-way and long range contacts. Without deriving a 3D reconstruction [15] showed that SPRITE not only recapitulates structural features found using Hi-C, but additionally identifies hubs of inter-chromosomal interactions that are arranged around the nucleolus and nuclear speckles. Importantly, these features were also detected using single-cell SPRITE assays (scSPRITE, [17]). Thus, it is of interest to determine what structural findings emerge from SPRITE-based 3D reconstruction in contrast with Hi-C-based reconstruction.

**Fig. 1:**
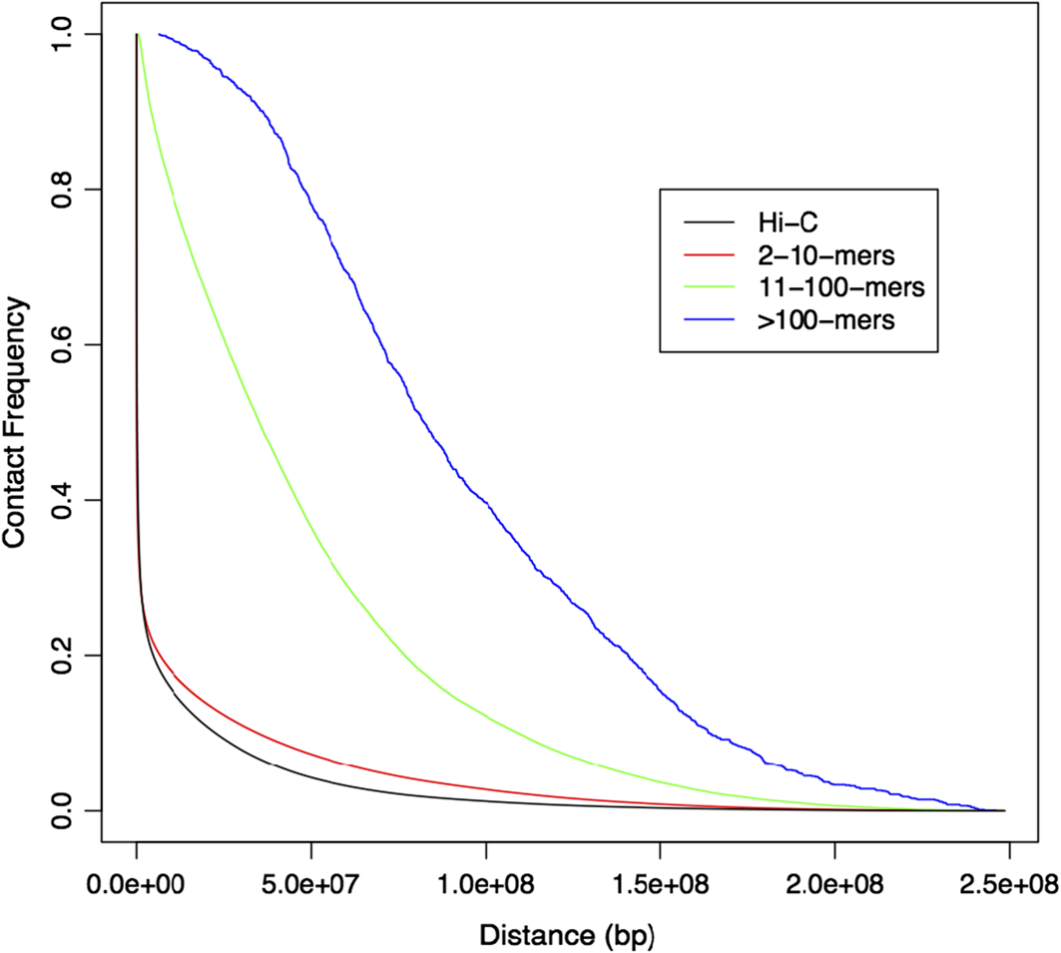
Contact frequency by maximum distance between reads for standard Hi-C [3] and SPRITE stratified by k-mer size

**Fig. 2:**
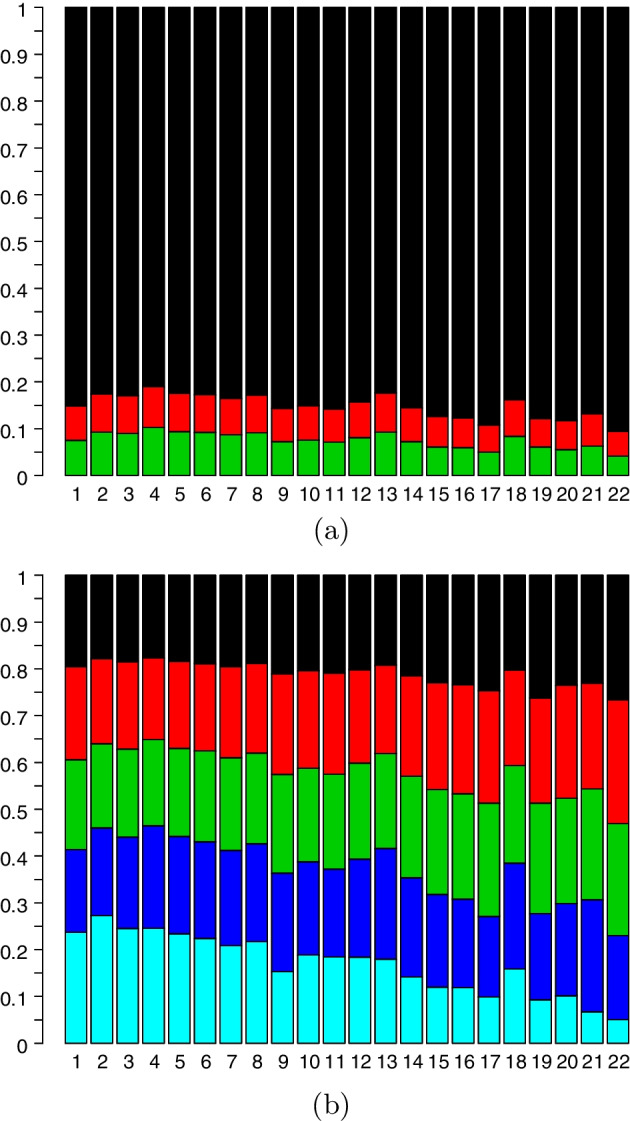
Barplots of **a** *k*-mer sizes (green > 3, red 3, black 2) and **b** distances (quintiles with first quintile black and fifth quintile cyan) of *k*-mers by chromosome. The first quintile ranged from 18 to 27%, the second from 17 to 26%, the third from 18 to 24%, the fourth from 17 to 24%, and the fifth from 5 to 27%

**Fig. 3:**
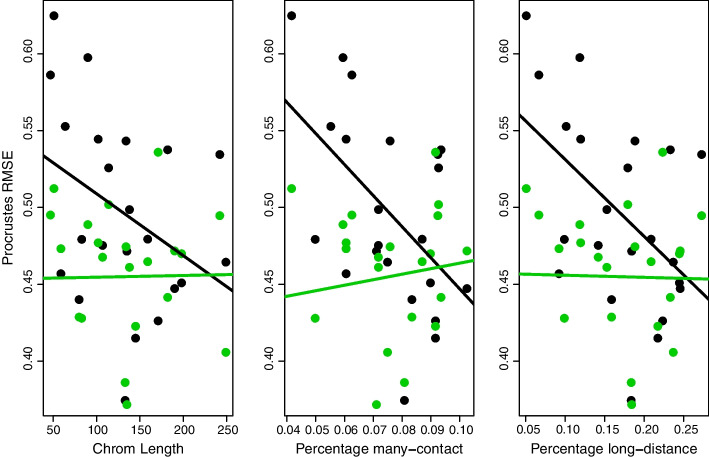
Procrustes RMSEs as a function of length, percentage many-contacts and percentage long-distance contacts. Least squares fits relate these features to RMSEs obtained using the N-W 3D reconstruction as target, with black and green designating aligned U-W and O-W 3D reconstructions, respectively

Figure 1, which recapitulates Fig. 3B from Quinodoz *and others* [15], provides further motivation for our work. By stratifying the genomic distance straddled by contacts according to degree of interaction it clearly demonstrates that high-order multi-way ($>10$) contacts broach appreciably greater distances than low-order (2–10) contacts, this latter stratum closely approximating Hi-C. Given these differences, and the presumed influence of long-range contacts on 3D reconstruction, we anticipate that differential weighting of contacts by their interaction degree will strongly impact resulting 3D configurations. Exploring and quantifying such relationships is the purpose of this work.

In pursuing this agenda we deliberately simplified the choice of reconstruction scope and algorithm. For the former, we only investigated intra-chromosomal reconstructions owing to the relative sparsity of Hi-C inter-chromosomal contacts. For the latter, we utilized a simple multi-dimensional scaling (MDS) routine with minimal parameter tuning. The reason we opted for such simplicity was to better facilitate comparisons between reconstructions based on the two assay types without added confounding.

For Hi-C-based deployment of MDS reconstruction techniques the first step is conversion of pairwise contacts to pairwise distances, this typically being achieved using power-law transformation [5, 18–21]. For SPRITE, converting multi-way contacts to pairwise distances is less immediate and investigation of differing strategies for so doing, described in Methods “Contact matrices"section, forms a central part of our work. These schemes allowed us to investigate the key differentiating factors (highly long-range and highly multi-way contacts) on attendant 3D reconstructions. It is anticipated that these factors, seemingly substantive contributors as suggested by Fig. 1, could significantly impact 3D reconstruction by anchoring more distal and complex folding. Possibilities for utilizing higher-order distances are addressed briefly in the Discussion.

Evaluating 3D reconstructions based on contact data is limited by the absence of gold standards. While recent, higher-resolution imaging modalities such as multiplex FISH [22] or in situ genomic sequencing (IGS, [8]), have facilitated accuracy assessment methods for 3D reconstructions ([23, 24] respectively), the absence of such image data on the same cells as SPRITE data precludes applying such approaches. Consequently, we appraise the role of multi-way contact weighting schemes, and the extent of long-range interactions and the degree of high-order contacts by comparing reconstructions across chromosomes where these latter features exhibit natural variation.

## Methods

### Data sources

We obtained SPRITE interaction data for the human lymphoblastoid cell line GM12878 from the Gene Expression Ominbus (GEO) repository GSE114242. As indicated above, we restricted analyses to intrachromosomal interactions for the twenty two autosomes. Specifically, we identified SPRITE “clusters” (“*k*-mers”)—the terms from Quinodoz et al. [15] for contacts of order *k*—with at least two intra-chromosomal contacts and with quality score MAPQ $\ge$ 30. These where then used to construct intra-chromosomal contact matrices for each of the autosomes.

In situ Hi-C [3] data from the GM12878 cell line were obtained from GEO repository GSE635625. We considered only the “primary” data series from this cell line. We utilized RAWobserved contact matrices for all autosomes with quality scores MAPQ $\ge$ 30.

**Fig. 4:**
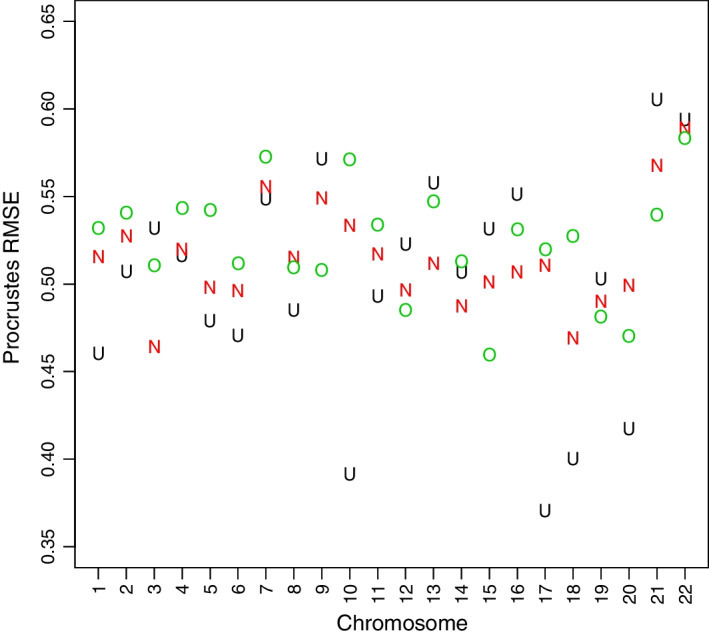
Procrustes RMSEs for a standard Hi-C reconstruction target compared to SPRITE reconstructions using U-W (black U), N-W (red N), and O-W (green O)

All analyses were performed at 25kb resolution (following binning contacts into intervals of corresponding genomic distance) in part to facilitate comparisons with respect to previously identified regions of interest. Multiple reads from the same SPRITE cluster in the same bin were only counted once. Therefore, the *k* in each *k*-mer was the number of bins that had at least one read. The exception was if all reads were in one bin, which were counted as 2-mers.

### Contact matrices

The Hi-C data readout is typically summarized by an $n \times n$ symmetric and non-negative matrix called a contact matrix that we denote by *C*. Each element $C_{ij}$ corresponds to the number of contacts between the $i\hbox {th}$ and $j\hbox {th}$ bins. As we have emphasized, SPRITE data features multi-way contacts rather than the strictly two-way (pairwise) contacts captured by Hi-C. While Hi-C data lends itself to immediate formation of *C*, with each pairwise contact between bins *i* and *j* incrementing $C_{ij}$ by one, this is not the case for SPRITE contacts as there are differing weighting possibilities for mapping multi-way contacts to their pairwise components [15]. Since we anticipated that highly multi-way contacts could exert a disproportionate influence on 3D chromatin configuration reconstruction, as motivated in the Background, we evaluated different schemes for mapping multi-way contacts to contact matrices.

The first approach we term “under-weighting” (U-W). It consists of adding 2/*k* to $C_{ij}$ for every pairwise interaction from a *k*-mer multi-way contact. This down-weighting was used (selectively) by Quinodoz et al. [15], following some reasoning surrounding minimally connected graphs, and was presumably favored because it more faithfully recapitulated Hi-C contact matrices than alternate weighting approaches. However, it is potentially detrimental to 3D reconstruction that might benefit from emphasizing multi-way and long-range proximities.

The second approach we call “neutral-weighting” (N-W). Here every pairwise interaction from a multi-way contact adds one to $C_{ij}$. The third approach we term “over-weighting” (O-W). Under this method every pairwise interaction from a *k*-mer adds $k(k-1)$ to $C_{ij}$. Thus, for a *k*-mer interaction the sum of U-W weights is 2, the sum of N-W weights is $k \atopwithdelims ()2$, and the sum of O-W weights is $k^2$.

Many approaches to normalizing Hi-C contact matrices have been advanced [25–29]. To prevent any normalization-dependent results, and because our analyses are purely comparative, our primary analyses do not utilize contact matrix normalization. However, we selectively explored the impact of a representative normalization scheme using the Hi-Corrector method [28].

### 3D reconstruction

As noted in the Background, we used multidimensional scaling to effect 3D reconstruction of individual chromosome configurations. MDS takes as input a (pairwise) distance matrix and outputs the 3D coordinates that best recapitulates these distances, the solution being prescribed to lie in 3D. There are several measures of ‘best recapitulates’ [18] and a variety of MDS algorithms. Here we utilize the mds function from the smacof R package that is based on a minimization-majorization algorithm [30]. We incorporated weights that are inversely proportional to distance [18], unweighted results (not shown) being similar. To obtain the input distance matrix from the respective contact matrices we applied a power-law transformation with index $-1/3$ [19], with, again, results being insensitive to alternative, previously used specifications ($-1.08$; [1]).

Akin to our handling of normalization we have opted to base primary analyses on a simple approach (multidimensional scaling, MDS) to 3D reconstruction that requires minimal tuning so as not to complicate conclusions surrounding competing multi-way contact weighting schemes. But, to ensure conclusions are not overly MDS-specific, we additionally utilized a second reconstruction method, PoisMS [31], that is based on principal curve metric scaling, which prioritizes a fundamental property of 3D chromatin architecture, namely, that its configuration constitutes a 1D smooth curve within the nucleus.

### Evaluating reconstruction differences

In order to compare competing 3D reconstructions, which are inherently coordinate system free, alignment thereof is necessary. Here we are interested in *reflection similarity shape*, under which two configurations that only differ by a reflection, rotation, translation and scaling are deemed equivalent. We effected such Procrustes alignment using the corresponding function from the vegan R package [32], from which we obtained the between-structure root mean square error (RMSE), our chosen measure of 3D reconstruction agreement. As a secondary method to evaluate the similarity between reconstructions, we utilized the generalized RV (GRV) test [33], which represents a generalized correlation coefficient applicable to 3D reconstructions and their attendant interpoint distance matrices. R software to perform the test was provided by the authors.

### Regions of interest

In addition to these global comparisons, we pursued some focal investigation of two specific genomic regions showcased by Quinodoz et al. [15, Figure 2] with a view to qualitatively assessing how contact matrices derived from the differing weighting schemes above compare in detecting previously identified features. The first region, Chromosome 6 (6p21-p22, 25.5Mb-28.0Mb), encodes 55 human histone genes and is highlighted since it illustrates SPRITE’s ability to detect multiple (here three) non-contiguous interacting genomic regions that skip intervening transcriptionally inactive segments. The second region, Chromosome 8 (8q24, 133.77Mb-134.57Mb) is highlighted as it displays how multiple loops may form higher-order interactions that simultaneously bring together distinct genomic regions, in this example three consecutive CTCF anchored loops.

## Results

SPRITE differs from Hi-C in capturing multi-way contacts and, as illustrated in Fig. 1, higher-order interactions straddle appreciably greater genomic distances than lower-order interactions. Accordingly, exploring the impact of ascribing differing weights to such *k*-mers and, in conjunction, providing the first 3D reconstructions utilizing SPRITE data is of interest.

We commence by characterizing the distributions of *k*-mers and genomic distances straddled by clusters across the 22 GM12878 autosomes. For display purposes we partition SPRITE *k*-mers into three groups according to there being two, three, or more than three contacts. A barplot of corresponding proportions by chromosome is presented in Fig. 2a that reveals the preponderance of two-mers but also shows inter-chromosomal variation. There are significant relationships , as determined by linear regression, between the percentage of many-contact *k*-mers, defined either as those with more than three ($p = 9{\text {e}}{-}4$) or more than 10 contacts ($p = 2{\text {e}}{-}5$), and chromosome length. Similarly, we examined the distribution of cluster extent (in terms of genomic distance) by chromosome by first determining overall distance quintiles and then plotting per chromosome proportions thereof in Fig. 2b. If we further define long-distance clusters as those belonging to the upper quintile, which roughly corresponds to those straddling more than 8Mb, we again observe significant association by linear regression between the number of long-distance *k*-mers and chromosome length ($p = 2{\text {e}}{-}10$).

**Fig. 5:**
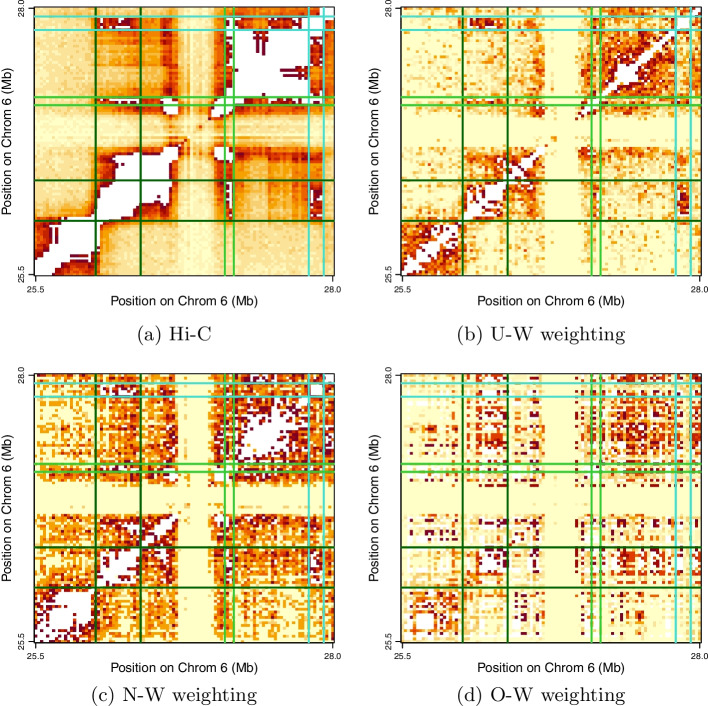
Heatmaps depicting contact matrices (of square root transformed counts) for the Hist1 region of Chromosome 6. Scales (not shown) differ according to assay type, weighting scheme and threshold (saturation) value. Darker pixels are for higher contact values, except white is for saturation. Regions of interest are defined by pairs of dark green, lime green and turquoise lines

**Fig. 6:**
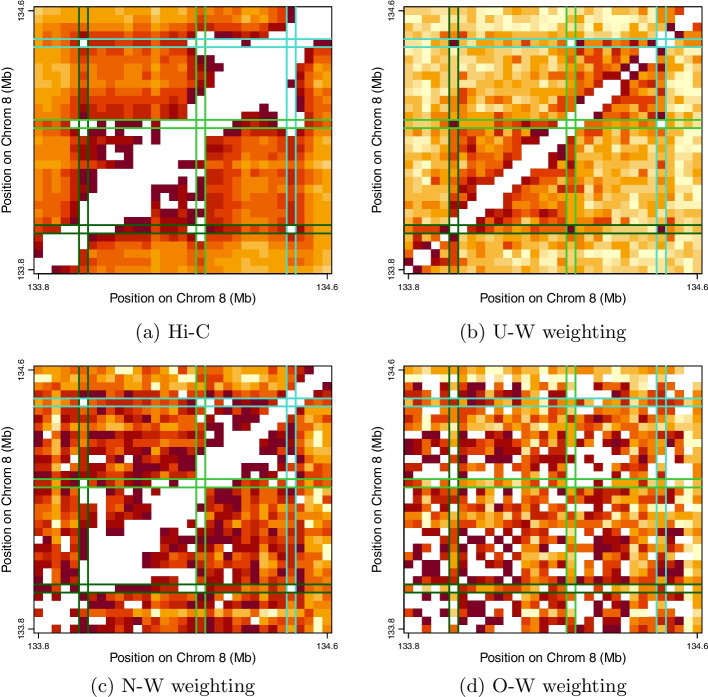
Same as Fig. 5. but for the contact matrices for the three consecutive CTCF anchored loops on Chromosome 8

Table 1 provides further detail by jointly summarizing *k*-mer counts according to genomic distance straddled, stratified by *k*-mer degree with strata selected to correspond to those in Fig. 1. While the preponderance of low-order *k*-mers is again evident, the contrasting impressions given by Table 1 and Fig. 1 is germane to subsequent findings surrounding 3D reconstruction as we discuss later.

**Table 1:** Cluster counts classified according to (genomic) distance extent and interaction degree

Distance (Mb)	2-mers	3-mers	4–10-mers	11–100-mers	> 100-mers
0–25	5,812,319	448,566	356,702	37,258	74
25–50	246,091	58,501	79,751	23270	228
50–75	146,326	30,175	41,402	14,828	299
75–100	90,422	16,177	20,616	8309	229
100–125	64,820	11,409	12,509	5020	181
125–150	40,446	7112	7288	3137	153
150–175	24,856	4339	4467	1887	103
175–200	13,693	2407	2257	996	64
200–225	7470	1275	1099	470	26
225–250	1885	331	339	156	20

We next obtained 3D reconstructions, using MDS, for each chromosome based on contact matrices derived from the U-W, N-W and O-W weighting schemes described in “Methods” section. To compare these 3D reconstructions we performed Procrustes alignment, with the (intermediary) N-W weight reconstruction serving as the referent (target) structure, and then computed root mean squared errors (RMSEs) relative to the U-W and O-W reconstructions. Results are displayed in Fig. 3. Based solely on RMSE magnitudes, it is apparent that 3D reconstructions using O-W weights scheme are closer to N-W weights than those obtained using U-W weights. For the latter N-W: U-W comparisons (black in Fig. 3) regression analyses reveal RMSE relationships with chromosome length ($\hbox {slope}=-0.0004$; $p = 0.09$), the percentage of many ($k > 3$) contact *k*-mers ($\hbox {slope}=-2.02$; $p = 0.01$) and the percentage of long (upper quintile $\approx 8$ Mb) distance *k*-mers ($\hbox {slope}=-0.50$; $p = 0.02$). However, after adjusting for chromosome length, many-contacts is barely not significant ($p = 0.07$) and long-distance is barely significant ($p = 0.03$). Similar results pertained to using alternative cutoffs to define many-contact *k*-mers ($k > 10$) and long-distance k-mers (upper decile). With the 3D reconstruction obtained using N-W weights again serving as the reference target, analogous regression analyses did not reveal any RMSE—feature relationships when comparing to aligned 3D reconstructions obtained using O-W weights (green in Fig. 3) as follows: length ($\hbox {slope}=0.00001; \hbox {p}=0.95$), percentage of many-contact $k-\hbox {mers}$ ($\hbox {slope}=1.5; \hbox {p}=0.59$), and percentage of long distance *k*-mers ($\hbox {slope}=-0.01; \hbox {p}=0.94$).

We examined whether our conclusion about the lack of impact of differing weights was dependent on our analysis choices. Specifically, we examined normalizing the contact matrices with Hi-Corrector [28], reconstructing the contact matrices with PoisMS [31], and comparing similarity with the GRV test [33]. The GRV test when comparing O-W and U-W to N-W has a p-value of 0 for every chromosome to the six digits reported, so the formal test was not useful, but the test statistic was. Similar to Procrustes, the GRV statistic, is higher (equivalent to Procrustes being lower) for the majority of chromosomes for O-W as compared to U-W. For normalized contact matrices, for the U-W comparison, neither chromosome length ($\hbox {slope}=-0.0001$; $p = 0.68$), nor percentage of many contact *k*-mers ($\hbox {slope}=-0.18$; $p = 0.53$), nor percentage of long distance *k*-mers ($\hbox {slope}=-0.48$; $p = 0.67$) is statistically significant. These same comparisons are similarly not significant for the O-W comparisons. For the PoisMS comparison, neither the U-W nor the O-W comparison is significant for any comparison. For example, for U-W the slope for length is 0.0006 ($p = 0.2$ ), for many contacts is 0.48 ($p = 0.26$) and for long contacts is 1.1 ($p = 0.52$).

From these findings it is apparent that assigning differing weights to SPRITE multi-way contacts did not have the anticipated impacts on 3D genome reconstructions: after adjustment for chromosome length, 3D reconstructions do not differ according to the extent of multi-way or long-range contacts. The reason for this result is clear from Table 1, namely, the dominance of low-order contacts. Such contacts, for which the different weighting schemes have the least effect, are so preponderant that they largely dictate the configuration of the respective MDS solutions.

As mentioned, the reason for Quinodoz et al. [15] selectively favoring the U-W down-weighting scheme was that it produced better agreement with Hi-C contact matrices by preventing large SPRITE clusters from disproportionately impacting (pairwise) contact frequencies. This determination was made in part visually and in part by appeal to Spearman correlations between the respective contact matrices. However, it has been argued [34] that such global correlation summaries are inappropriate for contact matrices. Accordingly, it is of interest to compare 3D reconstructions based on Hi-C with those deriving from SPRITE data under the differing weighting schemes. Resulting per chromosome RMSEs are displayed in Fig. 4. While it is the case that U-W weights generally (12/22 chromosomes) gave rise to 3D reconstructions closest to those from Hi-C (smallest RMSEs, often appreciably), the fact that this represents a bare majority implies that alternate weights cannot be discounted.

Finally, we revisit the showcased focal regions of interest [15, Figure 2], again with the goal of comparing the competing weighting schemes. These regions were originally highlighted since they illustrate SPRITE’s capacity to identify simultaneously occurring, high-order interactions, a hallmark of the assay. From the heatmaps depicting contact counts for the relevant segment of Chromosome 6 (Fig. 5) we observe that both U-W and N-W weighting highlight the appropriate sub-regions defining the simultaneous interaction, whereas O-W does not. Specifically, for the U-W and N-W weighting schemes, contact counts in the designated sub-regions exceed count totals in all equally-sized sub-regions at the corresponding genomic locus (following diagonal exclusion), while this is not the case for O-W. The same findings with respect to weighting scheme apply to identifying the three consecutive CTCF anchored loops that constitute a higher-order interaction resulting from simultaneously bringing together distinct genomic regions on Chromosome 8 (Fig. 6). From this we can conclude that under-weighting is not unique in being able to capture multi-way interactions. That such weighting possibly serves to best recover pairwise Hi-C contacts is not in and of itself a justification for its adoption.

## Discussion

Motivated by Fig. 1, we sought to examine the effect of differing approaches to counting the multi-way contacts generated in large numbers by SPRITE assays. As the figure illustrates, such contacts straddle relatively large genomic distances, implying that they may be particularly informative in capturing folding patterns not accessed by pairwise contact data, as provided by Hi-C assays. However, somewhat surprisingly, we found that for the different weighting schemes considered, there was little impact on attendant 3D reconstruction and, after adjusting for chromosome length, little association with the degree of multi-way contacts and/or extent of long-range contacts. Reconciliation of these findings came by way of simple accounting: despite there being numerous multi-way contacts, their numbers were dwarfed by the numbers of low-order contacts (see Table 1), with these findings being relatively unaffected by choice of weights.

The possibility remains that coercing SPRITE’s multi-way contacts to pairwise distances does not take full advantage of the power of higher-order interactions to anchor chromatin folds. *Tensors* provide a mathematically appropriate representation for multi-way contacts and definitions of tensor inner product, norm and distance generalize their vector analogs. Therefore, conversion of multi-way contact counts to tensor distances could follow a similar power-law transformation as was used here with (pairwise) Hi-C contacts. Then attendant reconstruction approaches, in particular tensor distance based multilinear multidimensional scaling [35], could be applied. Alternatively, higher-order distances as operationalized in the SPRITE context [13] could be utilized.

Further opportunities for additional work include utilizing SPRITE data to develop whole genome reconstructions for which existing methods for relative positioning of individual chromosome solutions [21, 36] could be used. Also, attempting 3D reconstructions based on single-cell SPRITE data could be pursued, thereby overcoming the problem that consensus structures based on bulk cell assays do not capture inter-cellular structural variation. The main challenge facing such reconstruction, also true for single-cell Hi-C, is the appreciable sparsity of single-cell interaction data: often $\sim 98\%$ of contact matrices are zeros. To address this challenge we are devising distribution-based MDS methods that allow for zero inflation [37].

## Conclusion

Long-range, multi-way contacts, as provided by SPRITE assays, do not appreciably impact 3D genome reconstruction, since the number thereof, while sizeable, is dwarfed by the number of pairwise contacts.

## Data Availability

The SPRITE data from [15] can be found at GEO under accession number GSE11424 (https://www.ncbi.nlm.nih.gov/geo/query/acc.cgi?acc=GSE114242). The standard Hi-C data from [3] can also be found at GEO under accession number GSE63525 (https://www.ncbi.nlm.nih.gov/geo/query/acc.cgi?acc=GSE63525).
